# 644. Outcomes and Predictors of Contracture in Hand Burn Grafting Through an Outpatient Database

**DOI:** 10.1093/jbcr/irag033.481

**Published:** 2026-04-06

**Authors:** Laura K Pezzopane, Laura Stephens, John H Loftus, Nicole P Bernal, Ariel Rodgers, Michael R Young, Nidhi D Aravapalli

**Affiliations:** The Ohio State Comprehensive Burn Center, Columbus, OH; The Ohio State Comprehensive Burn Center, Columbus, OH; The Ohio State Comprehensive Burn Center, Columbus, OH; The Ohio State Comprehensive Burn Center, Columbus, OH; The Ohio State Comprehensive Burn Center, Columbus, OH; The Ohio State Comprehensive Burn Center, Columbus, OH; The Ohio State Comprehensive Burn Center, Columbus, OH

## Abstract

**Introduction:**

Hand burns present unique challenges due to the hand’s complex anatomy and functional demands. Full thickness burns often require skin autografting, but contractures remain a common complication, leading to long-term morbidity. Despite surgical and rehab advances, no standardized national database tracks outcomes across specialties. Our objective is to develop a database tracking outcomes of surgically treated hand burns at our institution, identifying factors linked to successful recovery versus contracture development. The goal is to inform real-time improvements in surgical technique, rehabilitation, and follow-up care.

**Methods:**

A retrospective chart review was conducted for patients who underwent hand autografting for third-degree burns between 2023–2025. Data included demographics, burn characteristics, graft type, surgical service, rehab compliance, discharge location, and social determinants of health. Outcomes measured: contracture incidence/severity, hypertrophic scarring, functional status, secondary procedures, and predictors of contracture.

**Results:**

Of 365 admissions, 54 met inclusion criteria (surgical intervention without pre-existing hand contractures); 13 (24%) developed contractures. Burns involving both dorsal and palmar surfaces were present in 85% of contracture cases. Flexion contractures occurred in 53%. All contracture patients showed some level of rehab non-compliance. Social factors (e.g., substance use, transportation barriers, low literacy/income) affected follow-up in 8 of 13 cases. Graft type, surgical service, and palm grafting were not significantly associated with contractures.

**Conclusions:**

In developing a database through a retrospective review of outcomes after hand skin grafts, we found 24% of patients developed contractures. Burn characteristics and surgical approach did not show a pattern of contracture formation in this sample size. Contracture formation was influenced by postoperative and patient characteristics, particularly patient compliance. The palm tends to be an area that is left to heal and rarely grafted but was found to be the most common type of contracture. This database identifies the multidisciplinary aspect of hand burn recovery and identifies the characteristics that are higher risk based on outcomes. We identified elevated risk patients and injuries that can be used to focus post-operative care and discharge care plans on the patient's individual needs, including social determinants of health.

**Applicability of Research to Practice:**

A dedicated database that incorporates functional outcomes, multidisciplinary factors, and patient characteristics could directly inform practice by guiding individualized postoperative care, including splinting protocols, closer follow-up, and strategies to enhance adherence with rehabilitation.

**Funding for the study:**

N/A.

